# Comparative study of obstructive urolithiasis and its sequelae in buffalo calves

**DOI:** 10.14202/vetworld.2017.156-162

**Published:** 2017-02-08

**Authors:** Yasmin H. Bayoumi, Noura E. Attia

**Affiliations:** Department of Animal Medicine (Internal Medicine), Faculty of Veterinary Medicine, Zagazig University, Egypt

**Keywords:** buffalo calves, clinical, laboratory, obstructive urolithiasis, ultrasonography

## Abstract

**Aim:**

The present work was designed to study the incidence of obstructive urolithiasis and to apply comparative diagnosis to urine retention cases.

**Materials and Methods:**

A total of 78 non-castrated buffalo calves aging 3-11 months were included in this study, 68 calves were admitted to the Veterinary Teaching Hospital, Zagazig University, Egypt, during the study period with a history of anuria, and they were classified into three groups; intact bladder group (19 calves), uroperitoneum group (45 calves), and ruptured urethra group (4 calves). 10 apparently healthy calves were used for comparison. On the basis of history, clinical, laboratory, and ultrasonographic findings diagnosis was achieved.

**Results:**

There was a marked increase in the incidence of obstructive urolithiasis in winter season, especially in winter months of 2016. Calves within the age of 3-4 months and 6-8 months were mostly affected. Inappetence to anorexia, restlessness or depression, and abdominal distension were the most observed signs in the diseased calves. Laboratory findings revealed hemoconcentration and a significant increase in blood urea nitrogen and serum creatinine levels in all diseased groups. Hyperproteinemia, hypocalcemia, and hyperphosphatemia with electrolytes imbalance were recorded in the uroperitoneum group. Ultrasonographically, distended urinary bladder with distal acoustic enhancement revealed obstructive urolithiasis with intact bladder while anechoic fluid in abdominal cavity indicates uroperitoneum.

**Conclusion:**

On the basis of all findings, calves with intact bladder were in superior condition than those with a ruptured urethra and both were better than those with uroperitoneum.

## Introduction

Economically, urolithiasis is an important disease of domestic animals. The difference between urolithiasis and obstructive urolithiasis is an important one. Obstructive urolithiasis means the formation of calculi in the urinary tract with a urinary blockage. Simple urolithiasis has a relatively little importance, but obstructive urolithiasis has a significant life-threatening consequence [1]. Factors such as diet, sex, age, breed, genetic makeup, season, water intake, soil, mineral, hormone, and urinary tract infections play a key role in the genesis of urolithiasis [2].

Urethral obstruction causes distension of the urinary bladder, and as the bladder continues to distend, the animal exhibits signs of pain reactions until perforation of the urethra or rupture of bladder occurs. Such complications usually occur within 2-3 days if the obstruction is not relieved, on perforation and rupture, the animal may not show signs of discomfort anymore [3]. Obstructive urolithiasis with the intact bladder is a critical condition that requires immediate surgical intervention for correction of the condition to avoid complications [4]. Rupture of the bladder in turn, results in a flow of urine into the peritoneal cavity with subsequent uroperitoneum. If the urethral rupture occurs, the urine will leak into the connective tissue of the ventral abdomen and prepuce leads to an obvious fluid swelling. This may result in a severe cellulitis and toxemia [5].

Obstructive urolithiasis results in a series of abnormalities that arise from a failure of excretory process and accumulation of waste products in the body with fluid and electrolyte disturbances. The disturbances were qualitatively similar but quantitatively differ between steers with a ruptured bladder and ruptured urethra [6].

Diagnosis of obstructive urolithiasis is based on history, clinical, laboratory, and ultrasonographic findings. History and clinical examinations remain the first steps while differential diagnosis needs to be refined by laboratory aids. Sonography is a non-invasive imaging technique and inexpensive method for diagnosis of urolithiasis [7-9]. If used correctly, the sonogram will make the diagnosis more accurate, easier and faster. Thus, this study was aimed to study the incidence and to apply comparative diagnosis to cases of obstructive urolithiasis and its sequelae; ruptured bladder and ruptured urethra in male buffalo calves.

## Materials and Methods

### Ethical approval

The study protocol was approved by the Ethics Committee of Egyptian Veterinary Medicine Authority.

### Animals

The study was conducted between November 2014 and May 2016. All calves came during this period were included in the study even subjected to surgery or not. 68 non-castrated buffalo calves aging 3 to 11 months were be enrolled during the study period. The calves were presented to the Veterinary Teaching Hospital, Zagazig University, Egypt, with a history of anuria and signs of discomfort or depression. Beside ten apparently healthy buffalo calves were used as a control.

### Clinical examination

At the time of admission, clinical examination was thoughtfully performed; a thorough history regarding age, location, feeding, duration of retention, and observed signs was taken. Preliminary general examination and physical examination to all calves were done, and vital signs were monitored according to Jackson and Cockcroft [10].

### Laboratory analysis

Two blood samples were collected from all animals by jugular vein puncture. 1 ml of blood was transferred into ethylenediaminetetraacetic acid coated tubes for measuring packed cell volume %, and 5 ml of blood was collected for serum separation. Serum total protein, blood urea nitrogen, creatinine, calcium, and inorganic phosphorus levels were determined spectrophotometrically by standard procedures using (Diagnostic Zrt. Commercial kits) provided by Biomerieux, Egypt. Serum sodium and potassium were determined by the flame photometric method. Urine samples were also collected during surgical intervention in plastic cups and diagnostic urine test strips (Boehringer Mannheim, Germany) were used for rapid urinalysis.

### Ultrasonographic examination

Transabdominal ultrasonography of the urinary bladder was performed on standing position using a 3.5 or 5 MHz real-time B-mode sector transducer (SSD-500, Aloka, Tokyo, Japan). Transrectal ultrasonography of the pelvic urethra was performed by 6-8 MHz real-time B-mode linear transducer (SSD-500, Aloka, Tokyo, Japan) according to Braun [9].

### Treatment

Catheterization of the urethra was the initial trial in intact bladder cases. All diseased calves were treated surgically either by urethrostomy or tube cystostomy according to Amarpal *et al*. [11] except 5 calves; 3 calves had bad prognostic signs and unfit for applying surgery and 2 calves their owner refuse surgical intervention instead of a good condition.

### Statistical analysis

Analyses of variance with Duncan’s *post-hoc* test was used to determine the significance. The results were expressed as means±standard deviation. p<0.05 was selected as the criterion for statistical significance.

## Results

**Figure-1:**
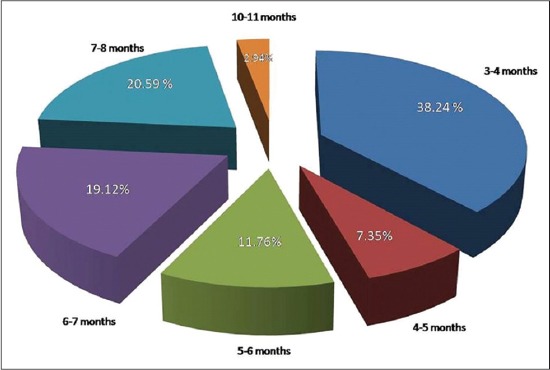
Age of diseased calves that enrolled during the study period.

**Figure-2:**
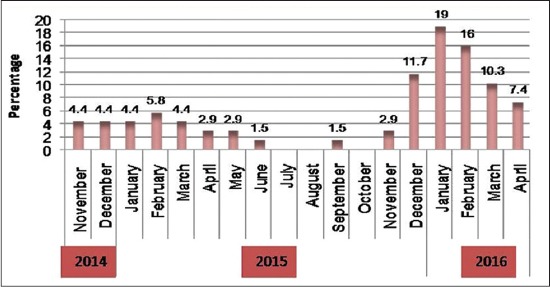
Monthly distribution of obstructive urolithiasis during the period of the study.

After inspecting files of veterinary teaching hospital clinic during the study period, and all cases suffering from urolithiasis admitted to the clinic between November 2014 and May 2016 were examined, we found the high incidence was in buffalo calves than cow calves and rams. All the diseased calves were uncastrated, and the highest incidence of the disease was recorded in young calves aged 3-4 months and 6-8 months (Figure-1). The diseases were more frequent in extreme winter, not in summer with the highest incidence in winter months of 2016 (Figure-2). It is worthy to mention that, rupture of the bladder was the common complication in buffalo calves while rupture of the urethra is less frequent.

On the basis of all findings, calves with obstructive urolithiasis were conventionally classified into three groups (Table-1).

**Table-1:** Classification of diseased calves that incorporated in the present study.

Groups	Number of calves Total=68	Age (month)	Anuria (day)
Intact bladder group	19	3-10	1-2
Uroperitoneum group*	45	3-8	3-7
Ruptured urethra group	4	3, 8, 10 and 11	3-4

* Uroperitoneum group includes 12 calves with intact bladder and 33 with a ruptured bladder

### Clinical findings

**Figure-3:**
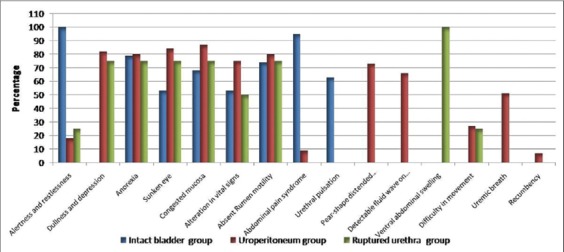
General and clinical examination of calves with obstructive urolithiasis.

Results of general and clinical examination of the diseased calves (in percent) were illustrated in Figure-3. Anorexia, varying degrees of dehydration “sunken eyes” and congested conjunctival mucosa were observed in all diseased groups. Regarding the alterations in the vital signs, in intact bladder group, the vital signs were slightly altered (42%) followed in an increasing order by ruptured urethra group (50%), and greater alterations were recorded in uroperitoneum group (75%). Of uroperitoneum group, the recumbent calves recorded more severe and critical alterations than other groups “dullness and depression, distended abdomen, detectable fluid wave on percussion, uremic breath and recumbency.”

In intact bladder group heart, respiration rates and body temperature were within the normal range or slightly increased. Tachycardia and polypnea together with variable body temperature were recorded in remaining groups. Subnormal body temperature (36-37.5°C) was recorded in 20% of uroperitoneum group. Variability in heart rate (tachycardia with weak heart beats or bradycardia) and periodic character of respiration with subnormal body temperature were the predominant alterations in recumbent animals.

**Figure-4:**
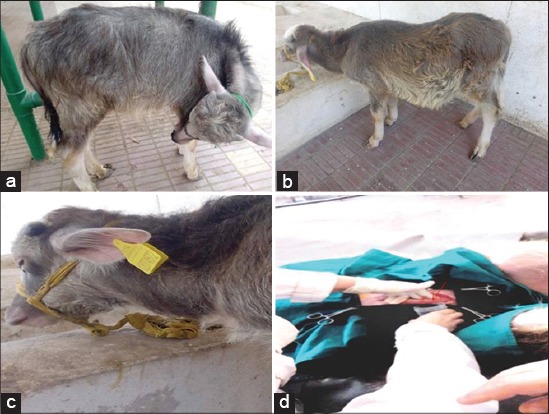
Buffalo calf, showing abdominal pain syndrome and restlessness (a). Buffalo calf with distention of abdomen, side view (b) signs of dullness and depression (c). The flow of urine from abdomen during the surgical intervention (arrow) in uroperitoneum cases (d).

Calves with intact bladder showed a syndrome of abdominal pain (100%) with kicking at the belly, restlessness (Figure-4a) and frequent unsuccessful attempts to urinate accompanied by straining, grating of the teeth and tail switching. Urethral pulsation was recorded in 63% of intact bladder cases. Concerning uroperitoneum group 82% of cases showed dullness and depression (Figure-4c), uremic breath (51%), pear shape distension of abdomen (73%) as showed in Figure-4b and difficulty in movement (27%). Detectable fluid wave on ballottement of the abdomen was mostly observed in the uroperitoneum group (66%), recumbency was recorded in 7% of uroperitoneum group. Regarding ruptured urethra group, 100% of cases showed ventral abdominal swelling caused by seepage of the urine under the skin that may extend to thorax with cellulitis which may be severe enough to affect the movement of the calf (25%).

### Laboratory analysis

Table-2 summarizes the result of laboratory analysis in healthy and diseased calves. Hemoconcentration was recorded in diseased calves with the highest hematocrit value in uroperitoneum group. Hyperproteinemia was clearly observed in uroperitoneum and ruptured urethra groups. Significant increase in serum urea and creatinine in all diseased groups was recorded, with the greatest increase in uroperitoneum and ruptured urethra group respectively. Uroperitoneum group had significantly lower serum calcium and higher serum phosphorus levels than other groups. Electrolytes imbalance, hyponatremia, and hypochloremia were recorded in uroperitoneum and ruptured urethra groups.

**Table-2:** Means and SD of laboratory values in healthy and diseased calves.

Parameters	Healthy calves (n=10)	Calves with obstructive urolithiasis		

		Intact bladder group (n=15)	Uroperitoneum group (n=37)	Ruptured urethra group (n=5)
Hematocrit (%)	30.80±1.92^d^	35.00±2.24^c^	42.80±3.56^a^	39.00±1.58b
Total protein (g/dl)	7.06±0.34^b^	7.00±0.37^b^	8.56±0.53^a^	7.52±0.34b
BUN (mg/dl)	10.60±4.22^d^	64.00±1782^c^	147.40±20.07^a^	104.00±13.87b
Creatinine (mg/dl)	1.02±0.39^d^	3.04±0.53^c^	7.48±1.37^a^	5.08±1.01b
Calcium (mg/dl)	10.42±0.63^a^	10.40±0.71^a^	8.90±1.04^b^	9.48±0.49ab
Phosphorous (mg/dl)	4.41±0.43^b^	4.76±0.44^b^	6.40±0.78^a^	4.87±0.67b
Sodium (mmol/L)	143.60±2.30^a^	142.00±1.58a^b^	130.20±4.21^c^	139.00±2.24b
Potassium (mmol/L)	4.72±0.54a^b^	4.40±0.64^b^	5.24±0.27^a^	5.24±0.34a
Chloride (mmol/L)	101.60±3.65^a^	100.60±4.72^a^	80.20±4.32^c^	86.20±3.96b

Different superscripts in the same row indicate a significant difference at P<0.0. SD=Standard deviations

### Ultrasonographically

**Figure-5:**
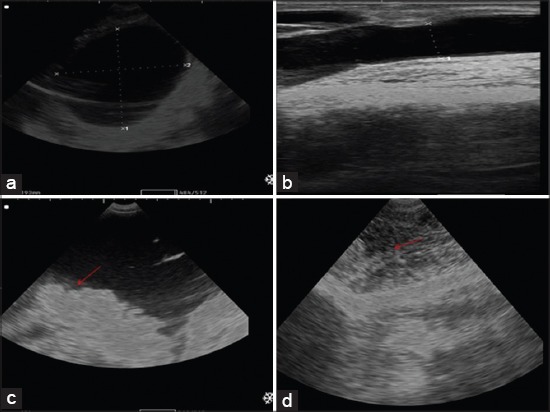
Transabdominal ultrasonogram of distended intact bladder. Notice, anechoic urine with distal acoustic enhancement (a), distended urethra transrectally (b). Ultrasonographic picture of uroperitoneum with ruptured bladder cases (c). Notice floating of viscera and omentum in fluids (arrows). Ultrasonographic finding of ventral swelling showed infiltration of anechoic urine (arrow) and extensive cellulitis (d).

Severely distended urinary bladder with anechoic urine with distal acoustic enhancement could be detected transabdominally (Figure-5a) in intact bladder group coupled with dilated urethra with anechoic urine transrectally (Figure-5b).

Distended urinary bladder with clear contour coupled with variable amounts of anechoic fluid in the abdomen was the ultrasonographic picture for an intact bladder with uroperitoneum cases. While uroperitoneum with ruptured bladder group revealed large amounts of homogeneous anechoic fluids, sometimes hypoechoic fluids (Figure-5c) involving the abdomen with floating of the viscera and omentum. Large amounts of fluids were released in the abdomen during the surgical intervention (Figure-4d). Ruptured bladder may be visualized transrectally and it does not always empty completely. It may also appear as collapsed balloon with thickened wall. In ruptured urethra group, the ultrasonographic picture of the urinary bladder and urethra were nearly similar to intact bladder group but with less pressure on both bladder and urethra. Sonogram of ventral abdominal swellings revealed infiltrated anechoic urine in subcutaneous tissue of ventral abdomen causing cellulitis (Figure-5d).

## Discussion

Because of the high incidence of obstructive urolithiasis in male buffalo calves in Egypt jointly with economic losses and caresses condemnation from uremia [12]. This study aimed to apply some comparative aspects that help in the diagnosis of obstructive urolithiasis and its sequelae.

Similar to our findings, Makhdoomi and Gazi [3] recorded a significantly higher incidence of obstructive urolithiasis in buffalo calves than cow calves. While Amarpal *et al*. [13] recorded high incidence of the disease in caprine, followed in decreasing order by cattle and canine. High incidence of the disease recorded between the age of 3 and 4 months could be attributed to movement of the calf to feedlot after weaning. While between the age of 6-8 months may be owed to keeping the animals on high concentrates low roughage ration. The previous record stated that the maximum numbers of cases were between 2 and 4 months of age followed by the animals of 4-6 months, 6-8 months and 8-10 months of age [14]. Limited water intake in very cold weather with high concentrates in ration may be a contributing factor behind the high incidence of the disease in winter months. In contrast to the present study, high incidence of obstructive urolithiasis was recorded in the extreme winter and summer [5].

In the present study, no correlation was recorded between castration and obstructive urolithiasis. And rupture of the urethra was less common than rupture of the bladder. Two from four cases of the ruptured urethra were occurred after excessive rubbing of the preputial region by the owner to promote urination, this might be an aid in perforation of the urethra by existence stone.

In fact, the general health condition of diseased calves was mainly related to the duration of illness and the status of urinary bladder and urethra, plainly with the amount of the urine intra-abdominal or subcutaneously. In general, calves with intact urinary bladder were in superior condition than those with the ruptured urethra and both were better than the uroperitoneum group and this may be related to metabolic changes associated with uroperitoneum. Grave disturbance in health condition was contemporary with the longer duration of anuria (5-7 days). The delayed admission of diseased calves to the clinic may be due to lack of attention of the owner or to the time taken for diagnosis and treatment trials. In the present study, days of retention mostly give a tentative diagnosis to the cases, history of anuria for 1-2 days mostly indicates intact bladder while anuria for 3 days or more indicates complications, a similar observation was recorded by Singh *et al*. [5]. It is worthy to mention that, a case of the bladder rupture occurred after 1 week from the treatment of hypomagnesemia in a calf. Hence, excess or imbalance minerals intake is incriminated in the occurrence of the disease [15].

Regarding the alterations in vital signs of diseased calves, Tiruneh [16] and Khan *et al*. [17] recorded similar findings. Tachycardia and polypnea could be attributed to dehydration, pain, and stress. Progressive systemic disturbances with accumulation of toxic metabolic waste products may be contributing factors [18]. Subnormal temperature recorded in 20% of uroperitoneum group could be attributed to retention of metabolic wastes and resulting toxemia [19]. Periodic respiration observed in recumbent calves is a bad sign caused by the effect of uremia on respiratory center [20]. Syndrome of abdominal pain recorded in all intact bladder cases could be owed to distension of the bladder with increased pressure in stretching receptor in its wall. Clear urethral pulsation was felt in 63% of intact bladder cases at the ischial region especially with the simultaneous rubbing of the preputial area to motivate efforts for urination [11]. Urethral pulsation is a good prognostic indicator for the existence of intact bladder.

In uroperitoneum group - with or without rupture of the bladder - calves appeared normally till the uremia developed, in which the animals exhibited the signs of dullness and depression. Relief of pain observed in ruptured bladder cases or reduction of pain in uroperitoneum with intact bladder cases and ruptured urethra groups could be owed to the seepage of the urine from the bladder and urethra, and subsequently, decrease the pressure on the stretch receptor in the bladder wall. Severe distention of the abdomen (pear shaped distention) caused by the release of large amounts of urine into the abdominal cavity. The distention may be to the point that calves were reluctant to move and prefer standing position to avoid the pressure on the diaphragm and lungs [5]. Calves with ruptured urethra may also exhibit difficulty in movement especially when diffused and extensive cellulitis occurred which may extend to the thorax. Uremic breath and interrupted breathing together with subnormal temperature were critical changes in recumbent animals and considered bad prognostic indicators and death follow.

Laboratory examination is a very useful tool to get insight into the animal health problems. In this study, laboratory examination plays an important role in grouping the diseased calves. Hemoconcentration and hyperproteinemia recorded in diseased groups are parallel with the degree of clinical dehydration due to shifting of the fluid into the peritoneal cavity.

A broad clarification to all biochemical changes recorded in the present study could be explained according to Radostits *et al*. [1] and Donecker and Bellamy [6] who stated that, in contrast to plasma, urine is usually hypertonic and it had a low concentration of sodium and chloride and high concentrations of urea, creatinine, potassium, and phosphate and so solutes redistribution occurs from high concentrations to low concentrations.

Significant increase in serum urea and creatinine levels was the classical findings in all diseased calves, measuring serum urea and creatinine is the first step for diagnosing the defect in excretion of metabolic wastes from the body and post-renal uremia might be a sequelae to obstructive urolithiasis. Sharma *et al*. [21] owed the increased levels of serum urea and creatinine in intact bladder group to decreased glomerular filtration rate as a result of back pressure on the kidneys. Higher serum urea and creatinine concentrations in uroperitoneum group could be owed to the movement of urea and creatinine from peritoneal cavity (high concentration) to the interstitial and intravascular compartment (low concentration). Similar results were recorded by Sharma *et al*. [22]. Larger absorptive surface area of the peritoneum than the subcutaneous area where the urethral perforation occurs and/or rapid calling for the veterinarian after observing the visible distention at ventral abdominal wall might be the possible reasons behind the difference in levels of serum urea and creatinine between the ruptured urethra and uroperitoneum groups. Elevated serum phosphorous level was clearly observed in the uroperitoneum group. Of uroperitoneum group, the highest levels of serum phosphorous was recorded in recumbent calves. Hence, serum phosphorus levels could be used as laboratory prognostic indicator in diseased calves [6]. Phosphorus retention causes a secondary hypocalcemia via increase calcium excretion in the urine [1].

Hyponatremia and hypochloremia could be attributed to the movement of sodium and potassium from interstitial compartment to the peritoneal cavity and vice verse hyperkalemia. Hyponatremia and hyperkalemia together with detectable hypocalcemia were responsible for skeletal muscle weakness and myocardial asthenia.

Urinalysis of diseased calves showed variation in color from yellow, amber to red color, turbid with slight to strong ammonia odor and more alkaline. Different degrees of proteinuria, erythrocytes, leukocytes and epithelial cell was detected in urine. Similar findings were recorded by Suresh Kumar *et al*. [23] proteinuria might be due to renal damage, which may cause passage of large protein molecules into urine. Epithelial cells, leukocytes, and erythrocytes detected in urine may be owed to the inflammatory reactions occurred by uroliths.

Ultrasonographic examination has considered as an increasingly valuable diagnostic tool that helps in decision-making. In this study, ultrasonography played a basic role in differentiating between the sequelae of obstructive urolithiasis and it emerged as the primary diagnostic imaging technique in diagnosis of obstructive urolithiasis [3].

Transabdominal ultrasonographic picture of the urinary bladder in the healthy animal was round to oval anechoic sac in the pelvis [7]. While in intact bladder group severe distention with distal acoustic enhancement could be detected. In uroperitoneum group, viscera and omentum were imaged floating in the fluid as previously observed by Abdelaal *et al*. [8]. Visualization of ruptured bladder greatly depends on the site of rupture; if ruptured bladders do not empty completely, the tear may be located dorsally at the neck of the bladder [1]. Increased the ruptured bladder wall thickness recorded in some cases could be greatly influenced by the degree of previous distention or degree of urinary bladder inflammation. The existence of hypoechoic fluid in the abdomen may be pointed to inflammatory products that indicate fair to poor prognosis dependently on vital signs alterations. All reported ultrasonographic findings were realistically seen during surgical intervention; the state of the bladder and urethra, urine in the abdomen and in ventral abdominal swelling. Hence, this study showed that the routine use of ultrasonograms during diagnosis procedures is a clinically useful technique.

## Conclusion

In summary, among the ruminants, the incidence of obstructive urolithiasis is high in male buffalo calves, especially in the winter season. Calves with uroperitoneum had more critical changes in clinical, laboratory and ultrasonographic findings than those with intact bladder and ruptured urethra cases.

## Authors’ Contributions

Both authors has planned and conducted the study equally. YHB wrote the manuscript. Both authors discussed the results. NEA revised the manuscript. Both authors read and approved the final manuscript.
